# Impaired Cerebral Autoregulation during Head Up Tilt in Patients with Severe Brain Injury

**DOI:** 10.1371/journal.pone.0154831

**Published:** 2016-05-11

**Authors:** Christian Gunge Riberholt, Niels Damkjær Olesen, Mira Thing, Carsten Bogh Juhl, Jesper Mehlsen, Tue Hvass Petersen

**Affiliations:** 1 Research Unit on Brain Injury Neuro Rehabilitation Copenhagen, Department of Neurorehabilitation/ TBI Unit, Rigshospitalet, Copenhagen, Denmark; 2 Department of Anaesthesia, Rigshospitalet, Copenhagen, Denmark; 3 Department of Neuroscience and Pharmacology, University of Copenhagen, Copenhagen, Denmark; 4 Department of Paediatrics, Copenhagen University Hospital Hvidovre, Hvidovre, Denmark; 5 Research Unit for Musculoskeletal Function and Physiotherapy, Department of Sports Science and Clinical Biomechanics, University of Southern Denmark, Odense, Denmark; 6 Department of Rehabilitation, Copenhagen University Hospital Herlev and Gentofte, Gentofte, Denmark; 7 Coordinating Research Centre, Bispebjerg & Frederiksberg Hospital, Frederiksberg, Denmark; Scuola Superiore Sant'Anna, ITALY

## Abstract

Early mobilization is of importance for improving long-term outcome for patients after severe acquired brain injury. A limiting factor for early mobilization by head-up tilt is orthostatic intolerance. The purpose of the present study was to examine cerebral autoregulation in patients with severe acquired brain injury and a low level of consciousness. Fourteen patients with severe acquired brain injury and orthostatic intolerance and fifteen healthy volunteers were enrolled. Blood pressure was evaluated by pulse contour analysis, heart rate and RR-intervals were determined by electrocardiography, middle cerebral artery velocity was evaluated by transcranial Doppler, and near-infrared spectroscopy determined frontal lobe oxygenation in the supine position and during head-up tilt. Cerebral autoregulation was evaluated as the mean flow index calculated as the ratio between middle cerebral artery mean velocity and estimated cerebral perfusion pressure. Patients with acquired brain injury presented an increase in mean flow index during head-up tilt indicating impaired autoregulation (*P* < 0.001). Spectral analysis of heart rate variability in the frequency domain revealed lower magnitudes of ~0.1 Hz spectral power in patients compared to healthy controls suggesting baroreflex dysfunction. In conclusion, patients with severe acquired brain injury and orthostatic intolerance during head-up tilt have impaired cerebral autoregulation more than one month after brain injury.

## Introduction

Severe acquired brain injury (ABI) causes major disability [1] and is often accompanied by a low level of consciousness in the early and sub-acute stage of rehabilitation[2]. As a consequence of the low level of consciousness, physical interventions in the early phases of rehabilitation primarily consist of passive mobilization, e.g. by head-up tilt (HUT) [3]. Early high-level mobilization is important for the functional outcome in patients with severe ABI, but may be limited by orthostatic intolerance [1, 4]. Orthostatic intolerance manifests as a rapid decrease in mean arterial pressure (MAP) or tachycardia, when the patient is mobilized [5, 6].

The cause of orthostatic intolerance is multifactorial, including a direct effect of the brain injury as well as prolonged bed rest [7–9]. In healthy subjects, cerebral blood flow is usually maintained at a relative constant level during HUT despite changes in MAP by adaptation of the cerebrovascular resistance, known as cerebral autoregulation [10, 11]. Clinically, a decrease in cerebral blood flow is usually associated with sweating, light-headedness, nausea, muscle weakness, and visual disturbances [12]. However, patients with severe ABI and low levels of consciousness often have spontaneous sweating periods whereby clinical signs of impaired cerebral blood flow can be difficult to interpret. It has been suggested that cerebral autoregulation is impaired in patients with severe ABI and that the severity of impairment is associated with long-term outcome [10, 13, 14]. However, these studies only assessed cerebral autoregulation in the supine position, and little is known about cerebral blood flow responses during HUT that adds additional stress to the cardiovascular system. If HUT affects cerebral blood flow, care should be taken when mobilizing these patients.

The aim of the study was to examine the cerebral autoregulation in patients with severe brain injury and low level of consciousness before, during, and after mobilization on a tilt-table.

## Material and Methods

Fifteen patients admitted to the Department of Neurorehabilitation/TBI unit at Rigshospitalet/Glostrup University Hospital, Denmark were consecutively enrolled from February 2014 to November 2014. This sample size was derived from data obtained by Hesse et al (2002) giving an estimated number of 15 subjects including a 20% dropout rate (β-level = 0.8 and α-level = 0.05) [15]. Inclusion criteria were: age ≥ 18 years, orthostatic intolerance during HUT (decrease in systolic or diastolic blood pressure of ≥ 20 mmHg and ≥ 10 mmHg, respectively, or increase in heart rate (HR) of ≥ 30 beats/min as defined by the European Society of Cardiology [12]), low level of consciousness and ABI. Low level of consciousness was defined as either vegetative state or minimally conscious state [2]. Vegetative state was defined as “complete unawareness of the self and the environment; it is accompanied by sleep-wake cycles with either complete or partial preservation of hypothalamic and brainstem autonomic functions” [16]. The minimally conscious state is characterized by inconsistent, but clearly discernible behavioural evidence of consciousness [2]. Exclusion criteria were: fractures, wounds, deep venous thrombosis, diabetes, or liver cirrhosis.

The study was approved by the regional ethical committee of the Capital Region of Copenhagen, Denmark (H-3-2013-024). Written informed consent was obtained from a legal proxy and the patient’s general practitioner before inclusion in the study. Fifteen healthy volunteers were recruited following provision of verbal and written informed consent.

### Experimental measurements

MAP was determined non-invasively by pulse-contour analysis using a continuous non-invasive arterial pressure system (CNAP Monitor 500 “HD”, CNSystems Medizintechnik AG, Austria (n = 5 patients; n = 14 healthy controls)) or Finometer (Finometer, Finapres Medical Systems, Amsterdam, the Netherlands) both of which uses photoplesmytographic continuous beat-to-beat measurement. Use of the CNAP monitor was discontinued due to malfunction. The cuff was kept at heart level during the experiment. Lead II electrocardiography (ECG) was used to measure heart rate and RR-intervals within the QRS-complex. Middle cerebral artery mean velocity (MCA Vmean) was evaluated by transcranial Doppler sonography (Multidop X; DWL, Sipplingen, Germany) with a 2 MHz probe placed over the temporal ultrasound window. Changes in MCA Vmean reflect those in cerebral blood flow [17, 18], as a stable diameter of the middle cerebral artery can generally be assumed [19]. MAP, ECG, and MCA Vmean were sampled at 1 kHz through an AD-converter (Powerlab, AD Instruments, Colorado Springs, CO, USA) and saved for further analysis using Labchart ver. 7.3 (Labchart, AD Instruments, Colorado Springs, CO, USA). Regional frontal lobe oxygenation (rScO_2_) was measured by near-infrared spectroscopy (NIRS) (INVOS 5100c Cerebral Oximeter^®^, COVIDIEN, Mansfield, Massachusetts, USA) [20]. The sensor was placed laterally on the forehead 2 cm above the margo orbitales superior. Changes in light absorption are considered to relate to haemoglobin oxygenation in blood vessels under the optode with some contribution from skin and skull [21]. NIRS data was sampled every five-six s (≈0.2 Hz). The transcranial Doppler and NIRS probes were placed on the patient´s least damaged hemisphere (right, n = 10). This decision was made by the treating physician supported by relevant imaging.

### Procedure

The subject was secured with straps on a tilt table with the feet against a footplate. Subjects rested for at least 30 min during instrumentation. Following this, 300 s of supine baseline recordings were made and the subjects were than tilted to 30°, 60° and 80° (head up) in 60 s intervals and stood for a maximum of 18 min at 80° tilt angle. If orthostatic intolerance occurred, the patient was immediately brought to the horizontal position and outcome measures were continued for a total of 30 min.

### Data analysis

Offline Data analysis was performed using MATLAB 2012b (Mathworks, Natick, USA). ECG data were pre-processed in Kubios HRV software (ver. 2.2, University of Eastern Finland, Finland) and inspected for artefacts. MAP, CPP_e_, MCA Vmean, HR and rScO_2_ was reported as baseline (300 s), the first 10 s during 30°, 60° and 80° HUT, the last 10 s of HUT (HUT0) and the post-tilt period (300 s after HUT0).

### Correlation between MAP and MCA Vmean

Autoregulation was analysed by calculating the mean flow Index (Mx) [13, 22] as the ratio of MAP and MCA Vmean. Two different calculations were performed: 1) 10 s averages using the raw MAP signal, and 2) 10 s averages corrected for the hydrostatic pressure gradient between the heart level and the cranial base referred to as the mean estimated cerebral perfusion pressure (CPP_e_). To account for the hydrostatic pressure gradient we estimated the distance from the level of the heart to the cranial base to be approximately 30 cm. A model was applied to continuously estimate the CPP_e_ at the level of the cranial base with a change in tilt angle of 3.75°/ s:

$$CPP_{e}=MAP_{heart\;level}-30\;cm\times 0.7\;\frac{mmHg}{cm}\;\times \;\text{sin}\left(tilt\;angle\right)$$

A Pearson’s product moment correlation was then calculated between 30 averages of 10 s data i.e. a window length of 300 s through the entire recording of 30 min with no overlap between windows. Correlation coefficient values close to zero indicate intact autoregulation, whereas values closer to 1 or -1 suggest impaired autoregulation [23]. Due to differences in the time spend in the upright position between patients we report Mx values for the 300 s of baseline, the first 300 s of HUT and the first 300 s after HUT (post-tilt). Time was added from the post-tilt period if patients had less standing time than 300 s and the following interval was moved accordingly. We report Mx values calculated using both MAP (Mxa) and CPP_e_ (Mxc). Note that Mxa and Mxc are equal in the supine position. Prior to the analysis, artefacts arising from head or arm movement or calibration of the Finometer were removed from the data by visual inspection.

### ECG analysis

A power spectral analysis of the RR intervals between successive heart beats (taken from the QRS complex of the ECG wave) was undertaken to investigate the magnitude of low (0.05–0.15 Hz) and high frequency (0.15–0.35 Hz) content of the RR intervals. Low frequency content is associated with the arterial baroreceptor regulation and a mix of parasympathetic and sympathetic branches of the autonomic system whereas the high frequency content reflects parasympathetic activity [24]. RR intervals from baseline and the first 300 s of HUT were then exported to MATLAB for further analysis. A cubic spline interpolation of 4 Hz was applied to the data in order to ensure equidistant sampling rate and a Fast Fourier Transformation (FFT) was applied to the data in order to estimate the spectral content of the RR intervals. A window length of 64 s with an overlap of 50% was used. Prior to the FFT analysis the RR intervals were normalised to have unit variance (divided by the SD) in order to compare spectral estimates between patients and controls. Pooled estimates of spectral power for the two groups were calculated using pooling algorithms available from Neurospec 2.0 (neurospec.org) [25]. Statistical comparison of pooled spectral estimates was performed by calculation of the log10 ratio and 95% confidence intervals between group estimates [26], also available from Neurospec 2.0 (neurospec.org).

### Statistical analysis

All data were analysed using STATA ver. 13.1 (StataCorp. 2013, College station, USA). Statistical significance was set at P < 0.05. The effects of HUT on MAP, CPP_e_, MCA Vmean, HR, rScO_2_, Mxa and Mxc were analysed by a repeated measure mixed effects model. Restricted maximum-likelihood estimation was used as default, with individual measures modelled as a function of group (patient or healthy), time (baseline at supine position, 30°, 60° and 80° HUT, HUT0 and post-tilt), and the interaction between group and time. Data are presented as mean and SE if not otherwise stated. Graphical representations of the results were performed in SPSS ver. 22 (IBM Corp. 2013, Armonk, NY).

## Results

One patient was excluded due to orthostatic tolerance leaving fourteen patients and fifteen healthy volunteers for further analysis (Table 1). For one patient post-tilt analysis was not possible because of short recording time and for one patient and one healthy control ECG recordings were not available due to apparatus malfunction. Thus, 13 patients were analysed for post-tilt and 13 patients were used in the power spectral analysis. There was a significant difference in age between the two groups (*P* = 0.003, *Mann-Whitney Wilcoxon test*). Patients suffered mainly from traumatic brain injury or cerebral haemorrhage (Table 1). Glasgow Coma Scale, Early Functional Ability Scale and Functional Independence Measure indicated that all patients suffered from severe brain injury, although only three of the patients were in a vegetative state.

**Table 1:** Subject characteristics.

	Patients (n = 14)	Healthy controls (n = 15)
Age [years] (IQR)	64 (49–69)	31 (27–59)
Male sex (%)	7 (50)	7 (47)
Traumatic brain injury (%)	8 (57)	
Cerebral haemorrhage (%)	5 (36)	
Anoxic brain damage (%)	1 (7)	
GCS (IQR)	9 (8; 13)	
MCS (%)	11 (79)	
EFA (IQR)	31 (20; 45)	
FIM (IQR)	18 (18; 18)	
Days since injury (SD)	41 ± 12	
Days in ICU (SD)	26 ± 9	

GCS: Glasgow Coma Scale; MCS: Minimally Conscious state; EFA: Early Functional Ability; FIM: Functional Independence Measure; ICU: Intensive care unit. Values are mean ± SD or median and IQR. Percentages presented in brackets.

Orthostatic intolerance presented as tachycardia in four patients and as a decrease in blood pressure in ten patients. The mean tilt-time for the patients was 348 (range: 61–989) s. Five patients were tilted down at 60° HUT due to decrease in blood pressure and thus, only nine patients are included in the 80° HUT results (Table 2). None of the healthy subjects experienced orthostatic intolerance during the experiment.

**Table 2:** Central and cerebral hemodynamics.

	Baseline _a_	HUT 30° _a_	HUT 60° _a_	HUT 80° _b_	HUT0 _a_	Post-tilt _c_
MAP [mmHg]						
PT	96 (3)*	92 (3)	87 (4) †	93 (4) †	84 (5) †	97 (4)
HC	79 (3)*	77 (4)	79 (4)	82 (4)	92 (4)	80 (3)
HR [beats/min]						
PT	95 (4)*	99 (5)	103 (5)	113 (8)	110 (7)	97 (5)
HC	62 (2)*	69 (3)	71 (3)	76 (4)	82 (3)	61 (3)
CPP_e_ [mmHg]						
PT	96 (3)*	82 (3)	70 (4) †	72 (4) †	65 (4) †	97 (4)
HC	79 (3)*	66 (4)	61 (4)	62 (4)	73 (3)	80 (3)
MCA Vmean [cm/s]						
PT	43 (3)*	40 (3)	35 (3)	36 (4)	36 (3)	41 (3)
HC	64 (3)*	64 (2)	60 (3)	56 (3)	55 (3)	60 (3)
rScO_2_ [%]						
PT	65 (2)	64 (3)	61 (3)	63 (2)	57 (3) †	63 (3) †
HC	70 (2)	70 (2)	69 (2)	67 (2)	68 (2)	75 (2)

HUT: Head-up tilt; PT: Patients; HC: Healthy controls; MAP: Mean arterial pressure; CPP: Cerebral perfusion pressure; MCA Vmean: Middle cerebral artery velocity; HR: Heart rate; rScO_2_: Near infrared spectroscopy determined frontal lobe oxygenation. Values in brackets are SE.

*denotes significant difference between patients and healthy controls at baseline (*P* < 0.001).

^†^ denotes between group difference in change from baselines (*P* < 0.05).

^a^: Fourteen PT and 15 HC;

^b^: Nine PT and 15 HC;

^c^: Thirteen PT and 15 HC.

The mixed effects analysis showed an overall significant group difference in MAP from baseline to HUT0 (*P* < 0.001) (Table 2) as the patient group had an orthostatic fall in MAP of 12 ± 3 mmHg, whereas the control group showed an increase in MAP by 13 ± 2 mmHg. HR increased from baseline to 60° and 80° HUT and HUT0 (*P* = 0.041, *P* = 0.001 and *P* < 0.001, respectively) in both groups, without differences in orthostatic change between groups from baseline to any other time point. There were no overall difference in HR between baseline and post-tilt (*P* = 0.728). When correcting for hydrostatic pressure in the standing position the CPP_e_ dropped significantly in both groups from baseline to 30°, 60° and 80° HUT (*P* < 0.001) and HUT0 (*P* = 0.003), with the patient group having a larger drop at the HUT0 compared to the healthy controls (31 ± 3 mmHg *vs*. 6 ± 2 mmHg, respectively). MCA Vmean decreased in both groups from baseline to 60° and 80° HUT and HUT0 and from baseline to post-tilt (*P* = 0.008, *P* < 0.001, *P* < 0.001 and *P* = 0.013, respectively), but orthostatic changes were alike between patients and controls. Similarly, rScO_2_ decreased from baseline to 80° HUT and HUT0 (*P* < 0.001 and *P* = 0.007) and from baseline to post-tilt (*P* < 0.001). S1 Fig. illustrates the timespan and differences for patients and healthy controls during a HUT procedure. It is noteworthy that the MCA Vmean appears to decrease more in the patient group than in in the healthy controls from baseline to 60° HUT with a trend towards a difference in the orthostatic change between the two groups (*P* = 0.065).

Mxa changed overall for patients and healthy controls from baseline to HUT (*P* < 0.001), but not from baseline to post-tilt (*P* = 0.621) (Table 3).

**Table 3:** Flow index (Mx).

	Baseline (300 s)	HUT (300 s)	Post-tilt (300 s)
Mxa			
PT	0.04 (0.07)*	0.35 (0.09) †	-0.02 (0.06)
HC	0.35 (0.07)*	-0.15 (0.10)	0.39 (0.08)
Mxc			
PT	0.04 (0.07)*	0.50 (0.09) †	-0.02 (0.06)
HC	0.35 (0.07)*	0.28 (0.07)	0.39 (0.08)

Mx: Correlation index; HUT: Head-up tilt; PT: Patient; HC: Healthy control; Values in brackets is SE.

*denotes significant difference between patients and healthy controls at baseline (*P* < 0.01).

^†^ denotes between group difference in change from baselines (*P* < 0.001).

Mxc increased from baseline to HUT for the patients and decreased for the healthy controls with no overall statistical difference from baseline to HUT in either group (*P* = *0*.454). The orthostatic change from baseline to HUT showed a significant difference in Mxa and Mxc between patients and healthy controls (*P* < 0.001). There were no significant difference in Mxc between baseline and post-tilt (*P* = 0.597).

### Subgroup exploratory analysis

Subgroup analysis of the patients with orthostatic hypotension (n = 10), showed an increase in the Mxa/c index from 0.09 at baseline to an Mxc of 0.59 during HUT, whereas patients with orthostatic tachycardia (n = 4) appeared to have a similar Mxc index of 0.27 during HUT as the healthy control group. Patients with postural tachycardia had a small increase in MAP (by 4 mmHg) during HUT while MAP decreased in the rest of the patients (by 19 mmHg). The orthostatic increase in HR appeared higher in the tachycardia group than in the rest of the patients and the controls (41 beats/min *vs*. 5 beats/min *vs*. 20 beats/min, respectively). The postural decrease in MCA Vmean was 5 cm/s in the tachycardia group compared to 8 cm/s in the patients with orthostatic hypotension and 9 cm/s in the healthy subjects. rScO_2_ decreased similarly in both the tachycardia and the orthostatic hypotension group (by 8% and 7%). Due to the small number of patients experiencing tachycardia, we did not perform statistical tests on these subgroups.

### Power spectral analysis of RR intervals

Power spectral analysis of the RR intervals in the healthy controls showed a peak in the ~0.1 Hz frequency band at baseline (S2A Fig) and no significant change within this frequency band during HUT (S2B Fig). Larger magnitudes at baseline *vs*. HUT are indicated by positive values and vice versa in the log/log comparison (S2C Fig). The patients did not show a well-defined peak in the ~0.1Hz frequency band in the supine position (S2D Fig) and yet the spectral power within this frequency band tended to decrease even further during HUT (S2E Fig) as indicated by the positive values the in the log/log spectral comparison (S2F Fig). Log/log comparison of spectral power at baseline and HUT showed significantly lower levels at ~0.1 Hz for the patients compared to the control group (S2G and S2H Fig). In addition, we found a significant decrease in the high frequency band during HUT in healthy subjects, which was not found for the patients.

## Discussion

This present study demonstrates impaired cerebral autoregulation in patients with severe ABI during HUT as Mxc increased in the patient group with concurrent reductions in MAP, CPP_e_, rScO_2_ and MCA Vmean during orthostasis. Previous studies have shown impaired cerebral autoregulation during supine rest in patients with traumatic brain injury or stroke [13, 23]. The higher Mxa/c value at baseline for the healthy controls is in accordance with data presented by Piechnik et al (1999) [27]. Reinhard et al (2003) also demonstrated an Mxc index close to 0.3 on stroke patients with low level of stenosis and 0.5 for patients with high level of stenosis [23]. The low Mxa/c for the patients in supine position in our study, most likely reflects an intact autoregulation in this position. Interestingly, the constant Mx for the healthy controls throughout the experiement signifies that the cardiovascular system is responding to the increased orthostatic stress introduced by HUT, whereas the patients did not show this reaction to HUT. Sato et al (2012) showed in healthy volunteers an orthostatic reduction in MCA Vmean of 9% during 60° HUT [11]. Other studies have found a decrease by 16% during lower body negative pressure [28, 29], which is similar to our results (16% and 13% in patients and healthy controls, respectively). Although there was no difference in the change of MCA Vmean for patients and healthy controls, the change in rScO_2_ and Mxc during HUT may be of clinical importance. Furthermore, the difference could have been accentuated if the patients were not lowered to supine as our protocol dictated. The slight increase in MAP, CPP_e_ and rScO_2_ at 80° HUT is due to the fewer patients included at this time point, since five patients developed orthostatic intolerance at 60° HUT. Heart rate variability at frequencies around 0.1 Hz represent baroreflex regulation and the reduction found in this frequency range in the patients points to an impairment of the baroreflex explaining the orthostatic intolerance as reflected in postural reduction in MAP. Markedly reduced low frequency (~0.1Hz) content in the RR interval signal in the patients both during HUT and at rest are in line with previous findings in both severe and mild cases of TBI as measured in the supine position [30].

The differences between patients and controls with respect to baseline MAP, HR, and MCA Vmean may partly be due to the difference in age between our two groups, since younger people often presents lower resting MAP and HR and higher MCA Vmean [31] and this may also be the case for Mx. However, it should be noted that previous studies failed to demonstrate any age-related changes in cerebral autoregulation as expressed by Mx values measured at rest [32, 33] and during HUT [34]. Further, the main purpose of including the control group was to compare postural changes in Mx and not for direct comparison of absolute values in the two groups. In addition, The Mx value is known to vary to some extent as a study by Ortega-Gutierez et al (2014) found variations between healthy subjects of similar magnitudes as ours [35].

Despite these significant findings, a number of limitations should be considered. MCA Vmean is a surrogate measurement of cerebral blood flow that assumes a constant diameter of the cerebral arteries. In studies of healthy volunteers this assumption is in all likelihood correct [36, 37]. However, it remains to be fully tested in patients with severe brain injury [19]. Along these lines it has recently been shown that around 27% of patients with traumatic brain injury experience vasospasms, defined as a cerebral blood flow velocity of more than 120 cm/sec[38]. Although the severity of vasospasms has been associated with the severity of cerebral autoregulation impairment [39], none of our patients experienced values of cerebral blood flow velocity of that magnitude. Moreover, it was not possible to directly measure CPP in our patients as none of the patients were instrumented with an ICP measurement device as part of their treatment at the department. We assume that the patients had a stable intracranial pressure but a sudden change in the parameter would inevitable affect the CPP. Lastly, further precision could have been achieved through recordings of end-tidal CO_2_, which affects CPP and MCA Vmean by approximately 4 mmHg /% [40].

NIRS was included to investigate if this could be used as a quick and effective bedside tool for evaluating cerebral blood flow in patients with severe brain injury. In general NIRS data reflects local brain tissue oxygenation and it is therefore also a surrogate measure of cerebral blood flow [21]. Although a difference between the time points and groups showed a significant change in rScO_2_, it is beyond this study to establish clinically valuable cut-off points to be used during tilt-table training. Moreover, limitations in the sampling frequency of the equipment (≈0.2 Hz) and the inability to synchronize NIRS with ABP/CBVF measurement limited the possibility of more sophisticated analysis of rScO_2_ [41, 42]. Future studies may elucidate the predictive capability of NIRS determined rScO_2_ and a denser array covering both the affected and non-affected hemisphere may yield better results.

Interestingly, the four patients that presented with tachycardia during HUT did not show an increase in the Mxc during HUT pointing to a preserved cerebral autoregulation. These four patients can explain a large part of the increase in HR on group level, since the other ten patients had almost no increase in HR during HUT. Although care should still be taken, the tachycardia patients might to some extent be able to compensate during tilt, which is supported by the change in MCA Vmean that appeared to be lower than that of the healthy volunteers (9% decrease vs. 13%, in the tachycardia patients and healthy controls respectively). The time since injury was similar for these four patients compared the remaining patients (46 vs. 39 days). Despite this notion, it clearly remains to be investigated whether the impaired regulation of cerebral blood flow observed in our study is due to the severity or localization of the brain damage or the extent of immobilisation (days in micro-gravity) or both. One previous study performed on patients with spinal cord injury using the same HUT protocol as ours found that patients with symptomatic orthostatic hypotension also had large decreases in MCA Vmean during HUT [43]. Moreover, haemodynamic changes have been found in healthy young men confined to bed rest for 20 days [44]. Therefore, a role of immobilization alone on the autoregulation cannot be ruled out.

Cerebral autoregulation have been suggested to recover within the first month after severe traumatic brain injury [10]. In the present study the mean time since injury was 41 days which indicates that cerebral autoregulation still may be impaired at later stages. Orthostatic intolerance and impaired cerebral autoregulation limits further mobilization of some ABI patients because of risk of hypo perfusion of the brain. Because of this, these patients are referred to further bed rest or mobilised with the risk of increasing the severity of the brain injury. Given the association between early and intensive mobilisation on functional outcomes [4] both situations are at risk of deteriorating the effectiveness of the rehabilitation. Therefore, treatment strategies to overcome this challenge should be considered.

## Conclusion

Patients with severe ABI and orthostatic intolerance demonstrate impaired cerebral autoregulation during HUT more than one month after ABI which limits mobilization. In addition, patients with severe brain injury differ from healthy controls with respect to postural reduction in blood pressure and rScO_2_. This is supported by the finding of impaired baroreflex sensitivity expressed through frequency analysis of HR variability. Further research is needed focusing on methods to stabilise blood pressure and cerebral hemodynamics in the upright position to enable more intensive rehabilitation of patients with severe ABI.

## Supporting Information

S1 Fig Change from baseline. Change in MAP, HR, CPP, MCA Vmean and rScO_2_ for patients and healthy controls. * denotes between group difference in change from baselines (*P* < 0.05).
(TIF)

S2 Fig Power spectral analysis. Pooled power spectral analysis of the RR intervals at baseline (A&D) and head-up tilt (HUT) (B&E) for patients (PT) and healthy controls (HC), respectively. Log10 ratio of pooled power spectra between baseline and HUT for the healthy controls (C) and the patients (F) and between healthy controls and patients at baseline (G) and HUT (H). Dashed lines denote no difference and solid lines denote lower and upper 95% confidence intervals. Low frequency content marked by grey shaded area.
(TIF)

S1 File Spectral analysis. All data used for spectral analysis of RR-intervals.
(ZIP)

S1 Table Subject characteristics. Excel file containing data from Table 1 and in text data.
(XLSX)

S2 Table Central and cerebral hemodynamics. Excel file containing data from Table 2 and in text data.
(XLSX)

S3 Table Flow index (Mx). Excel file containing data from Table 3 and in text data.
(XLSX)
