# Supplementary figures and images for: Impaired Cerebral Autoregulation during Head Up Tilt in Patients with Severe Brain Injury
Source: PLoS One. 2016 May 11;11(5):e0154831. doi: 10.1371/journal.pone.0154831 (PMC4864314; doi:10.1371/journal.pone.0154831)

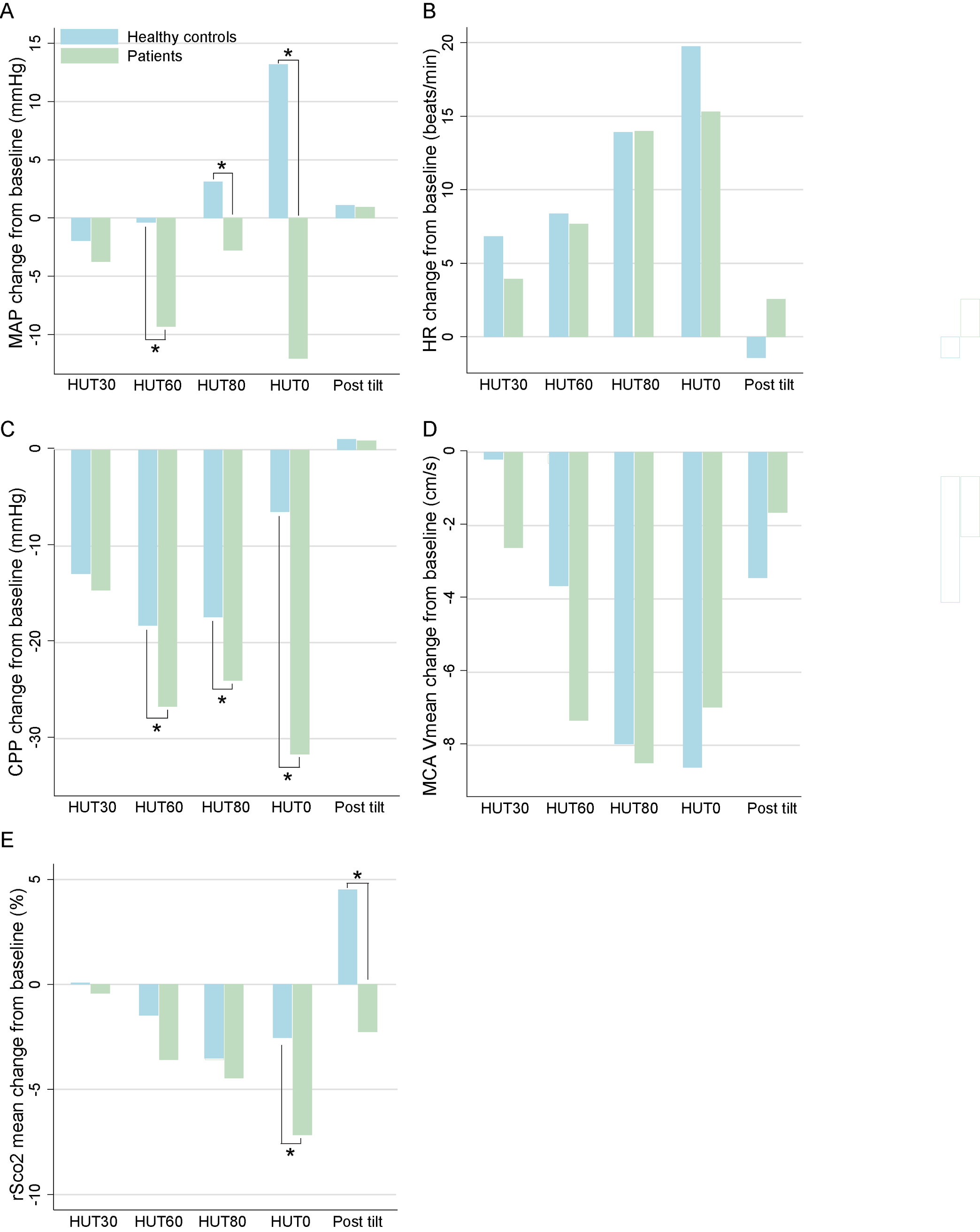

Supplement: S1 Fig — Change in MAP, HR, CPP, MCA Vmean and rScO2 for patients and healthy controls. * denotes between group difference in change from baselines (P < 0.05). (TIF) [file pone.0154831.s001.tif]

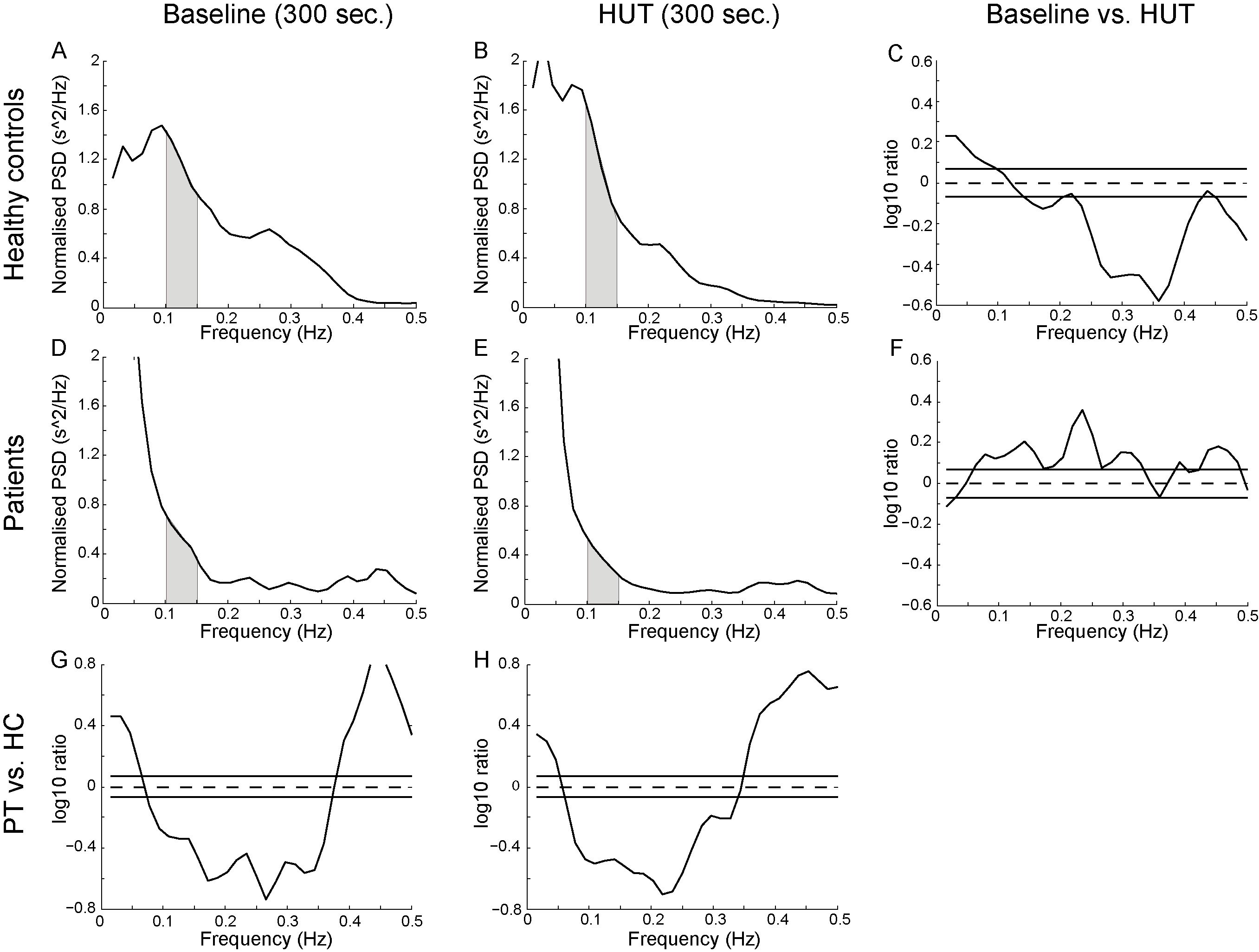

Supplement: S2 Fig — Pooled power spectral analysis of the RR intervals at baseline (A&D) and head-up tilt (HUT) (B&E) for patients (PT) and healthy controls (HC), respectively. Log10 ratio of pooled power spectra between baseline and HUT for the healthy controls (C) and the patients (F) and between healthy controls and patients at baseline (G) and HUT (H). Dashed lines denote no difference and solid lines denote lower and upper 95% confidence intervals. Low frequency content marked by grey shaded area. (TIF) [file pone.0154831.s002.tif]
