# Supplementary material for: Impaired Cerebral Autoregulation during Head Up Tilt in Patients with Severe Brain Injury
Source: PLoS One. 2016 May 11;11(5):e0154831. doi: 10.1371/journal.pone.0154831 (PMC4864314; doi:10.1371/journal.pone.0154831)
Supplement: S1 File — All data used for spectral analysis of RR-intervals. (ZIP) [file pone.0154831.s003.zip › S4 dataset/Spectral analysis.docx]

Spectral analysis in matlab was performed using Neurospec2.0 from neurospec.org

Please see documentation at homepage for further information on how to use the code.

Spectral analysis:

%%

data =(cell2mat(Res.HRV.Data.RRs)); % data from Kubious file

time = cell2mat(Res.HRV.Data.T_RRs) ; % time, used for interpolation

time = time-time(1); % relative timining

time_rs = 0.25:0.25:time(end); % resampled timing 4 Hz

yy = spline(time,data,time_rs)'; % spline interpolation

trig= (time_rs(1)*4:120:time_rs(end)*4-239)'; % trigger used for 50% overlap in spectral analysis

duration = ones(size(trig,1),1)*240; % segment length for

[f,t,cl,sc] = sp2a2_m1(1,yy,yy,trig,duration,4,8,' h2 t2 n'); % spectral analysis, uses Neurospec from neurospec.org

%%

f2= f;

f2(:,2)= 10.^f2(:,2); % f is log transformed data so inverse

figure()

plot(f2(1:32,1),f2(1:32,2)) % plot the data.

% f t cl and sc was saved for pooling analysis.

Pooling of data

clear all

[filename] = uigetfile( ...

{ '*.mat'}, ...

'Pick a file', ...

'MultiSelect', 'on'); % select saved files

%%

clearvars plf plv plf1 plv1

for x=1:size(filename,2)

load(filename{x})

if x==1

[plf,plv]=pool_scf(sc,cl); % call for first file to initiate pooling

else

[plf,plv]=pool_scf(sc,cl,plf,plv); % further iterations

end

end

[f2,t2,cl2,sc2] = pool_scf_out(plf,plv); % get pooled estimates

figure() % plotting

f22 = f2;

f22(:,2) = 10.^f22(:,2);

plot(f22(1:32,1),f22(1:32,2))

ylim([0 2])
